# Survival Rate of Breast Cancer in Eastern Mediterranean Region Countries: A Systematic Review and Meta-Analysis

**DOI:** 10.5334/aogh.2521

**Published:** 2019-12-04

**Authors:** Soheil Hassanipour, Ahmad Maghsoudi, Shahab Rezaeian, Morteza Arab-Zozani, Ali Mohammad Mokhtari, Elham Abdzadeh, Shirin Riahi, Shokrollah Mohseni, Hamid Salehiniya

**Affiliations:** 1 Gastrointestinal and Liver Diseases Research Center, Guilan University of Medical Sciences, Rasht, IR; 2 GI Cancer Screening and Prevention Research Center, Guilan University of Medical Sciences, Rasht, IR; 3 Student Research Committee, Shiraz University of Medical Sciences, Shiraz, IR; 4 Clinical Research Development Center, Imam Reza Hospital, Kermanshah University of Medical Sciences, Kermanshah, IR; 5 Social Determinants of Health Research Center, Birjand University of Medical Sciences, Birjand, IR; 6 Iranian Center of Excellence in Health Management, School of Management and Medical Informatics, Tabriz University of Medical Sciences, Tabriz, IR; 7 Social Determinants of Health Research Center, Mashhad University of Medical Sciences, Mashhad, IR; 8 Non-communicable Diseases Research Center, Alborz University of Medical Sciences, Karaj, IR; 9 Social Determinants in Health Promotion Research Center, Hormozgan Health Institute, Hormozgan University of Medical Sciences, Bandar Abbas, IR

## Abstract

**Background:**

Breast cancer (BC) is one of the main problems of public health around the world. As a consequence, survival rates are one of the most salient indicators for assessing the quality of cancer control and treatment programs.

**Objectives:**

The aim of this study is to evaluate the survival rate of breast cancer in the Eastern Mediterranean region at different periods of time.

**Methods:**

Medline/PubMed, ProQuest, Scopus, Embase, Web of Knowledge and Google Scholar databases until February 1, 2018. All observational studies (cross-sectional, case-control, and cohort) referring to the survival of breast cancer were included in the study. The heterogeneity and its value were examined by Cochran test and I^2^ statistics, respectively. Analysis of subgroups performed was based on geographical area and Human Development Index (HDI), using Stata 12 software.

**Findings:**

A total of 58 papers were analyzed. Based on a random effect model, the survival rates of breast cancer in different periods—1, 2, 3, 4, 5 and 10 years—were estimated at 93.9, 85, 79.8, 72.6, 69.2, 62.1 and 55.5 percent, respectively. The highest 10-year survival rate was in Iran (59.2%), and the lowest was observed in Bahrain (45%).

**Conclusions:**

Evidence suggests that about half of the patients in this area would die before 10 years survival, which is different from more developed countries. Also, high survival rates are associated with high human development index, which can help health policy-makers to better predict the outcomes of patients.

## Introduction

Breast cancer (BC) is the most common cancer among women, with about 1.7 million people affected every year. It is one of the major causes of cancer-related deaths in female population.

In recent decades, the incidence of BC has elevated in many developing countries as well as in developed countries. Despite this increasing trend in the incidence, the survival rate of patients has significantly improved in many developed countries. During the past decade, the rate of detection of BC has expanded, which is one of the factors affecting the survival of patients with this cancer [1,2,3,4,5]. Several studies have shown that surgical outcomes for benign and malignant diseases, especially in patients with BC, are improved in specialized centers. Early diagnosis and effective therapies for BC have led to an increasing number of survivors, thus the long-term health and well-being of survivors has been addressed by the scientific community.

Survival rates are one of the most important indicators for assessing the quality of cancer control and treatment programs. Over the past decades, the survival rate of advanced BC has improved significantly. According to studies, the five-year survival rate of women with BC over the years 1999 to 2005 was 90%, which increased by about 15% compared to 1975–1977.

Strong evidence suggests that the difference in the survival rates and incidence rates of malignancies such as BC may be related to social determinants of health. To classify and evaluate these factors, some indicators, such as socioeconomic status, level of education, occupation, income level, gender, race, ethnicity, religion, nationality, social class, and place of residence are used. The results of a study that investigated the survival of BC indicated that women in poor countries with a low educational level often have a lower survival rate. Factors such as race, ethnicity and state of affairs affect both access to BC care and its survival rate [2].

According to a study, the overall incidence and mortality of cancer in the Southeast Asian Region (SEARO) for men, and in the Eastern Mediterranean region (EMRO) for women, is lower than other WHO (World Health Organization) regions. Also, in terms of WHO regions, the Eastern and European Mediterranean districts had the highest rates of mortality of this cancer [6]. Considering the high rate of BC in the Eastern Mediterranean region, and the fact that survival rate is one of the most important indicators for assessing the quality of treatment, the present systematic review study aimed at evaluating the survival rate of BC in different periods of time (1, 2, 3, 4, 5, 7 and 10 years) for EMRO countries.

## Methods

The present study is a systematic review and meta-analysis of the estimation of BC survival in the EMRO. This study was designed and implemented in 2018 and reported based on the PRISMA (Preferred Reporting Items for Systematic Reviews and Meta-Analysis) checklist [7].

### Search strategy

We searched Medline/PubMed, ProQuest, Scopus, Embase, Web of knowledge and Google Scholar databases until February 1, 2018 without any limitation in language and publication years. The Selected keywords for international databases included: Neoplasm, Cancer, Carcinoma, Malignancy, Breast Cancer, Breast Neoplasms, Breast carcinoma, Breast Tumor, Cancer of Breast, Neoplasms of Breast, Survival, Survival Analysis, Survival Rate, Afghanistan, Bahrain, Iran, Iraq, Jordan, Kuwait, Lebanon, Oman, Pakistan, Qatar, Saudi Arabia, Syria, United Arab Emirates, Djibouti, Egypt, Morocco, Palestine, Somalia, Sudan, Tunisia, Libya, and Yemen. The collected data entered to the EndNote X7 software, then, duplicate articles were automatically deleted. It is worthwhile to mention here that two researchers individually screened the articles based on title, abstract and full text.

### Criteria for considering studies for this review

All of the observational studies (cross-sectional, case-control and cohort) that were published before February 1, 2018, which referred to the survival rate of BC in the countries of the EMRO, were included in the study. Studies that did not report the sample size or confidence interval of survival rate were not included in this study.

### Screening of studies

Initial research was conducted by two people (AM & ShR). Screening of studies, extraction of results and quality appraisal were done independently by two reviewers (EA & HS). Potential discrepancies were resolved by a third reviewer (SH).

### Data Extraction Form

We used a pre-piloted checklist for data extraction. This checklist includes items about the name of the author, the year of publication, the time period of the study, the country where the study was conducted, and the survival rate by year.

### Quality assessment

The Newcastle-Ottawa Quality Assessment Form was applied to evaluate the quality of included studies. This tool has three different parts, including selection (4 questions), comparability (1 question) and outcome (3 questions). Based on the final scores, studies divided into 3 categories Good (3 or 4 stars in selection domain, 1 or 2 stars in comparability domain and 2 or 3 stars in outcome/exposure domain), Fair (2 stars in selection domain, 1 or 2 stars in comparability domain and 2 or 3 stars in outcome/exposure domain) and Poor (0 or 1 star in selection domain, 0 stars in comparability domain or 0 or 1 stars in outcome/exposure domain).

### Statistical analysis

The heterogeneity of the included studies was evaluated by Cochran test (with significance level less than 0.1), and its composition by using I^2^ statistics. In case of heterogeneity, the random effects model was utilized with the photo-variance method, and in the absence of heterogeneity, the model of constant effects was applied. All analysis was performed using the STATA version 12 software.

### Additional analysis

Due to the heterogeneity of the studies, subgroups analysis was used. The indicator applied for this purpose was Human Development Index (HDI). The HDI is a relative measure of life expectancy, education, quality, education level, and in general, the living standards in human societies. This Index is estimated using the measure of welfare, especially among children and people of low age. These statistics can be applied to measure the development of countries, the impact of economic policies on living standards and the survival of breast cancer in each of the countries, to provide a clear indication of the breast cancer survival status in each country.

To investigate the potential source of heterogeneity, we conducted meta-regression based on year of study. Also, Egger’s test was used for assessing publication bias.

## Overall results

### Results

#### Results of the search

**Figure 1:**
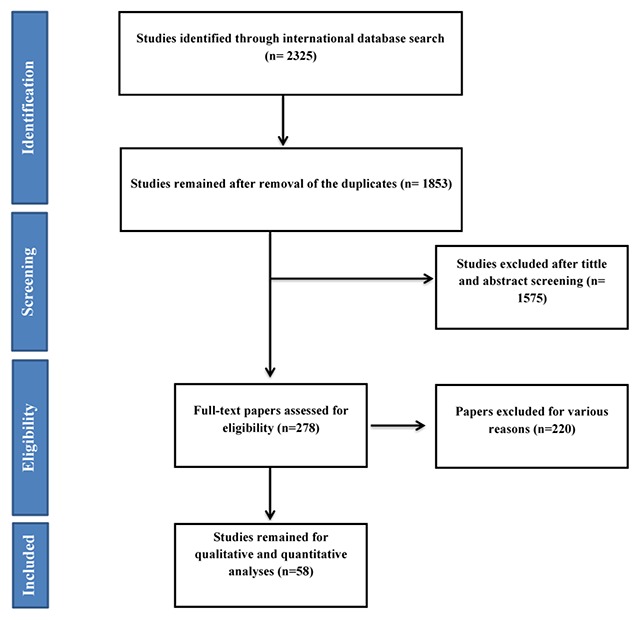
Flowchart of the included studies in systematic review.

After searching databases, 2,325 records were found. Of those, 1,853 records were entered for screening based on title and abstract after duplicate removal. Subsequently, reviewing the titles and abstract, 278 articles entered the next stage, at which point the full text of them was examined, and 58 progressed to the final step of analysis. It should be noted that the referenced articles were also reviewed to add related studies. In the screening stages of studies, they were excluded for a variety of reasons, which included unrelated topic, unassociated population, not reported sample size or confidence interval and duplicated studies. The flowchart of the included studies is presented in Figure 1.

#### Results of Quality Assessment

Based on our result, 41 studies had good quality and 17 studies had fair quality. The result of Quality Assessment is presented in appendix file 1.

#### Description of studies

Based on the geographical location of 58 included studies, 32 studies conducted in Iran, six in Saudi Arabia, four in Morocco [8,9,10,11], three in Tunisia, three in Pakistan, two in Oman, two in Bahrain, two in Jordan, two in Kuwait, one in Libya and one in Egypt. The summary characteristics of the included studies were shown in Table 1.

**Table 1:** Basic information of included studies.

Author, year	Time period	Country	Survival rate (%)						

			1	2	3	4	5	7	10

Al-Idrissi, 1992	1981–1981	Saudi Arabia					62		
Ibrahim, 1998	1985–1985	Saudi Arabia							55
Fakhro, 1999	1982–1982	Bahrain					68.8	57.3	39.31
Ahmed, 2002	1990–1990	Tunisia					50.5	50	
Al Moundhri, 2004	1996–1996	Oman					67		
Motawy, 2004	1993–1993	Kuwait					76		
Vahdaninia, 2004	1997	Iran					62		
Ghavam-Nasiri, 2005	1995–1995	Iran		80.4			47.7		
Ibrahim, 2005	1992–1992	Saudi Arabia					74		
Ravichandran, 2005	1994–1994	Saudi Arabia	93.9		79.2		59.6		
Babaei, 2005	1991–1991	Iran	94	89	84	76	61		
Rajaeifard, 2005	1993–1993	Iran	97		82		70		53
Mousavi, 2006	1998–1998	Iran					75		
Akbari, 2006	1996–1996	Iran					76.6		
Khanfir, 2006	1995–1995	Tunisia					57		
Yaghmaei, 2008	1991–1991	Iran	86.9		62		58		47
Heydari, 2009	2001–2001	Iran	97				67		45
Rezaianzadeh, 2009	2001–2001	Iran			76		58		
Sadjadi, 2009	2003–2003	Iran	92						
Abahssain, 2010	2003–2003	Morocco			80.6				
Arkoob, 2010	1997–1997	Jordan	91.6	80.1	70.2	65.8	59.3		
El Mongy, 2010	1999–1999	Egypt			96.4		91.4		
Al-Moundhri, 2011	2003–2003	Oman					78		
Fouladi, 2011	2003–2003	Iran					51		
Hamdan, 2011	1994–1994	Saudi Arabia	96.3		82.7		65.3		
Tarawneh, 2011	1997–1997	Jordan	93.1	82.7	74.33	68.7	64.2		
Movahedi, 2012	2001–2001	Iran					72		
Rahmani, 2012	2009–2009	Iran	97.3		87.02		75.72		
Rais, 2012	2007–2007	Morocco					76.5		
Vostakolaei, 2012	1999–1999	Iran					72		
Ziaei, 2013	1997–1997	Iran	96		86		81	79	76
Haghighat, 2013	1997–1997	Iran		96			87		
Fallahzadeh, 2014	2002–2002	Iran	95	86	82	76	70		
Fayaz, 2014	1999–1999	Kuwait			83.2		81.5		
Sedehi, 2014	2003–2003	Iran	83	71	67	59	51		
Fazeli, 2014	2007–2007	Iran	98	96	92	89	87		
Hamadeh, 2014	2000–2000	Bahrain	84				63		49
Faradmal, 2014	2004–2004	Iran					68		
Karimi, 2014	2006–2006	Iran					75		
Baghestani, 2015	1998–1998	Iran	93		75		52		
El Mistiri, 2015	2003–2003	Libya					60.6		
Jamshed, 2015	1995–1995	Pakistan					70		54
Mahmood, 2015	2000–2000	Pakistan					74.9		
Rampisheh, 2015	2001–2001	Iran	95	88	78	73	68		
Payandeh, 2015	2001–2001	Iran			82		72		64
Rejali, 2015	1994–1994	Iran							71.2
Derkaoui, 2016	2010–2010	Morocco					71.4		
Faradmal, 2016	2000–2000	Iran	90		73		62.5		
Kumar, 2016	1999–1999	Pakistan					75		
Mechita, 2016	2005–2005	Morocco	97.1		89.2		80.6		
Rahimzadeh, 2016	–	Iran	95.6		80.8		69.5		58.1
YektaKooshali, 2016	–	Iran					68.84		
Bakhshi, 2017	2010–2010	Iran	92						
Davoudi Monfared, 2017	2006–2006	Iran			87.8				
El Amine Elhadj, 2017	2004–2004	Tunisia		49.1	33.7				
Hosseinpour Feizi, 2017	2007–2007	Iran	98		88		82		
Najafi, 2017	2007–2007	Iran		96			88.1		

#### Heterogeneity

The results of the chi-squared test and the I^2^ index indicated that there was a considerable between-study heterogeneity. For one (I^2^ = 94.1%, P < 0.001), two (I^2^ = 98%, P < 0.001), three (I^2^ = 97.1%, P < 0.001), four (I^2^ = 96.2%, P < 0.001), five (I^2^ = 98.2%, P < 0.001) and 10-year survival rate (I^2^ = 92.7%, P < 0.001).

#### Synthesis of results

The articles were sorted according to the year of publication, and then analyzed by survival analysis of 1, 2, 3, 4, 5 and 10-year survival rate, based on a random effect model.

#### One-year survival

**Figure 2:**
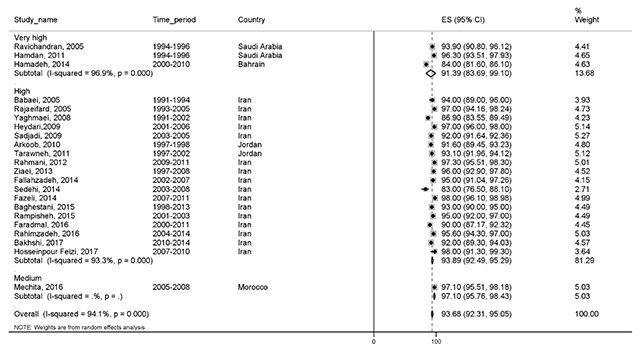
Forest plot of one-year survival rate of breast cancer in EMRO countries.

Twenty-two studies reported a one-year survival rate. The total number of samples was 32,884. The one-year survival rate was 93.6% (95% CI, 92.3–95) in the EMRO countries. One-year survival rate of BC based on HDI is shown in Figure 2.

#### Two-year survival

**Figure 3:**
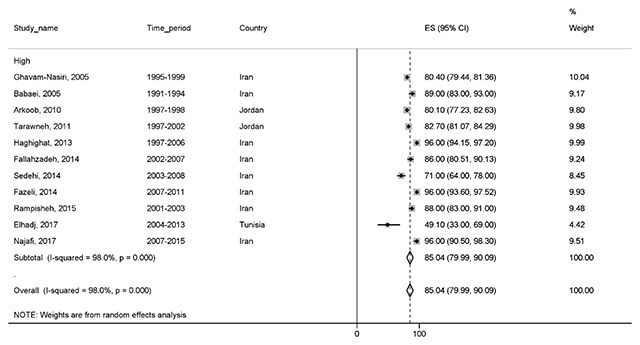
Forest plot of two-year survival rate of breast cancer in EMRO countries.

Eleven studies reported a two-year survival rate. The total sample size was 5,073 cases. The two-year survival rate was 85% (95% CI, 79.9–90). Two-year survival rate of BC based on the HDI is shown in the Figure 3.

#### Three-year survival

**Figure 4:**
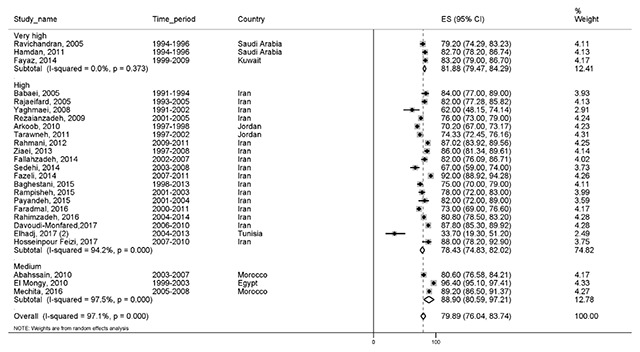
Forest plot of three-year survival rate of breast cancer in EMRO countries.

Twenty-five studies reported a three-year survival rate, with a total sample size of 34,225. The three-year survival rate was 79.8% (76–83.7) in EMRO countries. The results of three-year survival based on the HDI is illustrated in Figure 4.

#### Four-year survival

**Figure 5:**
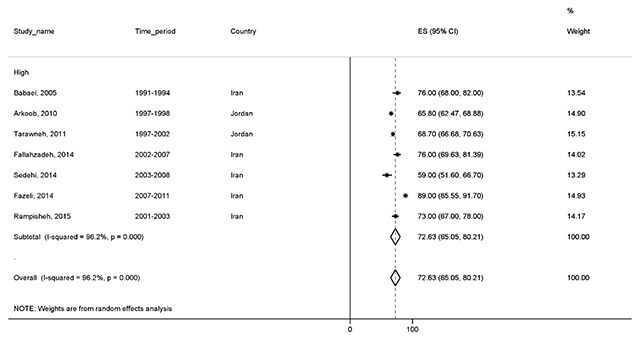
Forest plot of four-year survival rate of breast cancer in EMRO countries.

One of the lowest survival rates reported in all studies was the four-year survival rate. A total of seven studies with a total sample of 4,196 reported this result. Our findings showed that the four-year survival was 72.6% (95% CI, 65–80.2). The results of these studies are categorized by the HDI and are illustrated in Figure 5.

#### Five-year survival

**Figure 6:**
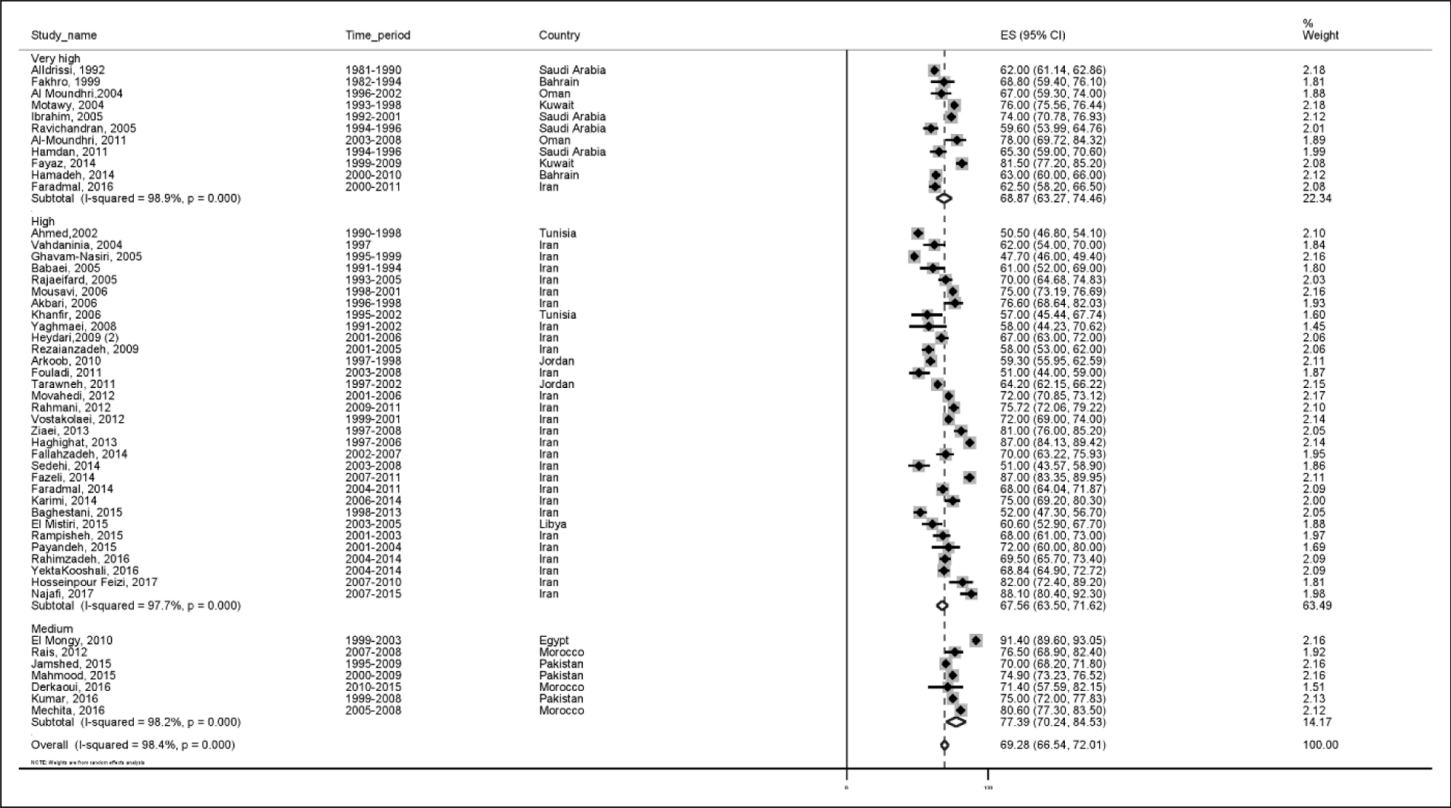
Forest plot of five-year survival rate of breast cancer in EMRO countries.

The highest reported survival rate was five-year survival rate. A total of 50 studies with a total sample size of 94,041 reported this statistic. The five-year survival rate was 69.2% (95% CI, 66.5–72). The results of the five-year survival rate by HDI are shown in Figure 6.

#### Ten-year survival

**Figure 7:**
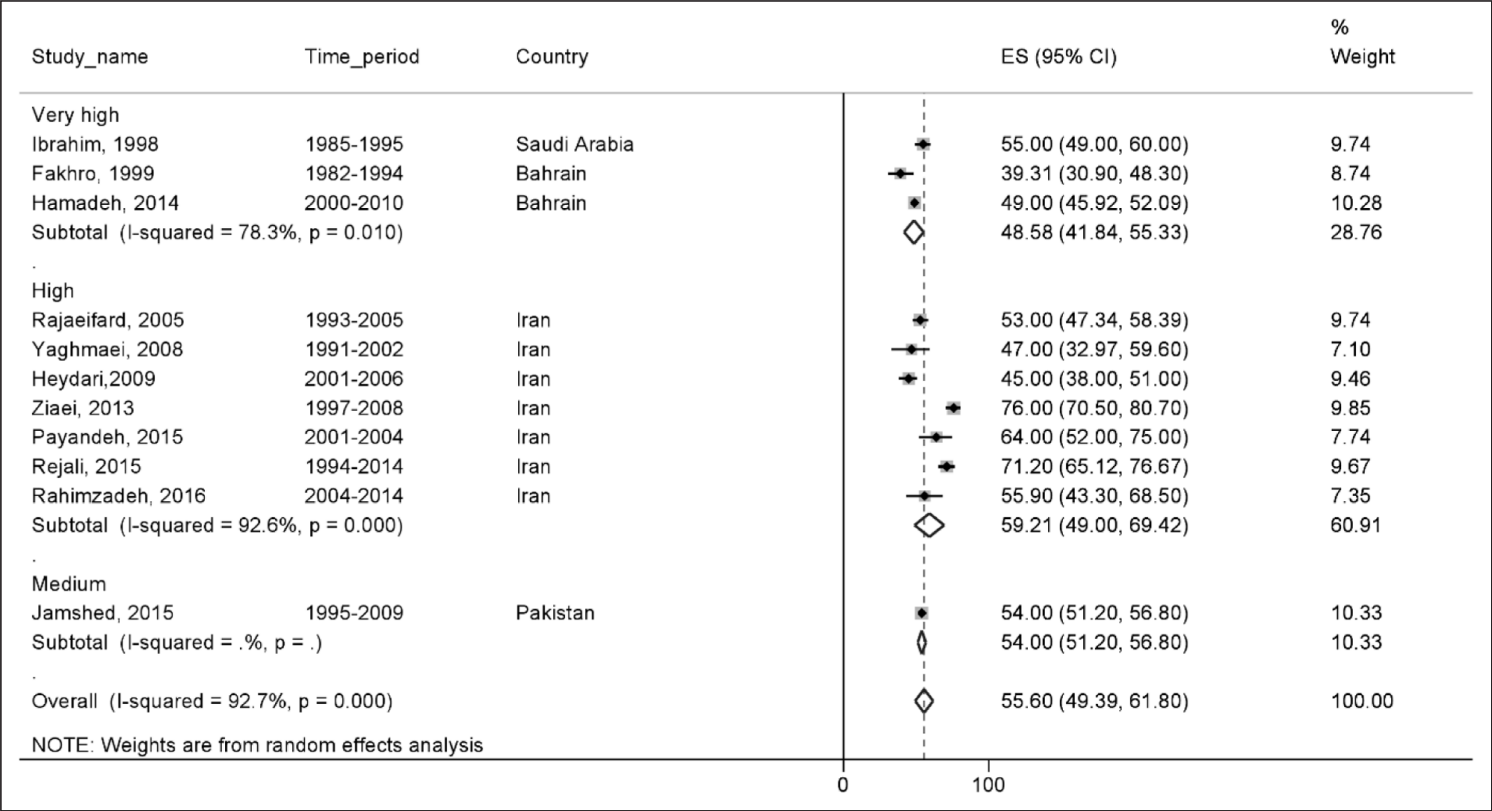
Forest plot of ten-year survival rate of breast cancer in EMRO countries.

A total of 11 studies, with a total sample size of 28,785, reported this survival rate. Based on the results, the 10-year survival rate was 55.5% (95% CI, 49.3–61.8). Ten-year survival of BC by HDI is shown in Figure 7.

#### Survival of breast cancer in EMRO countries

**Figure 8:**
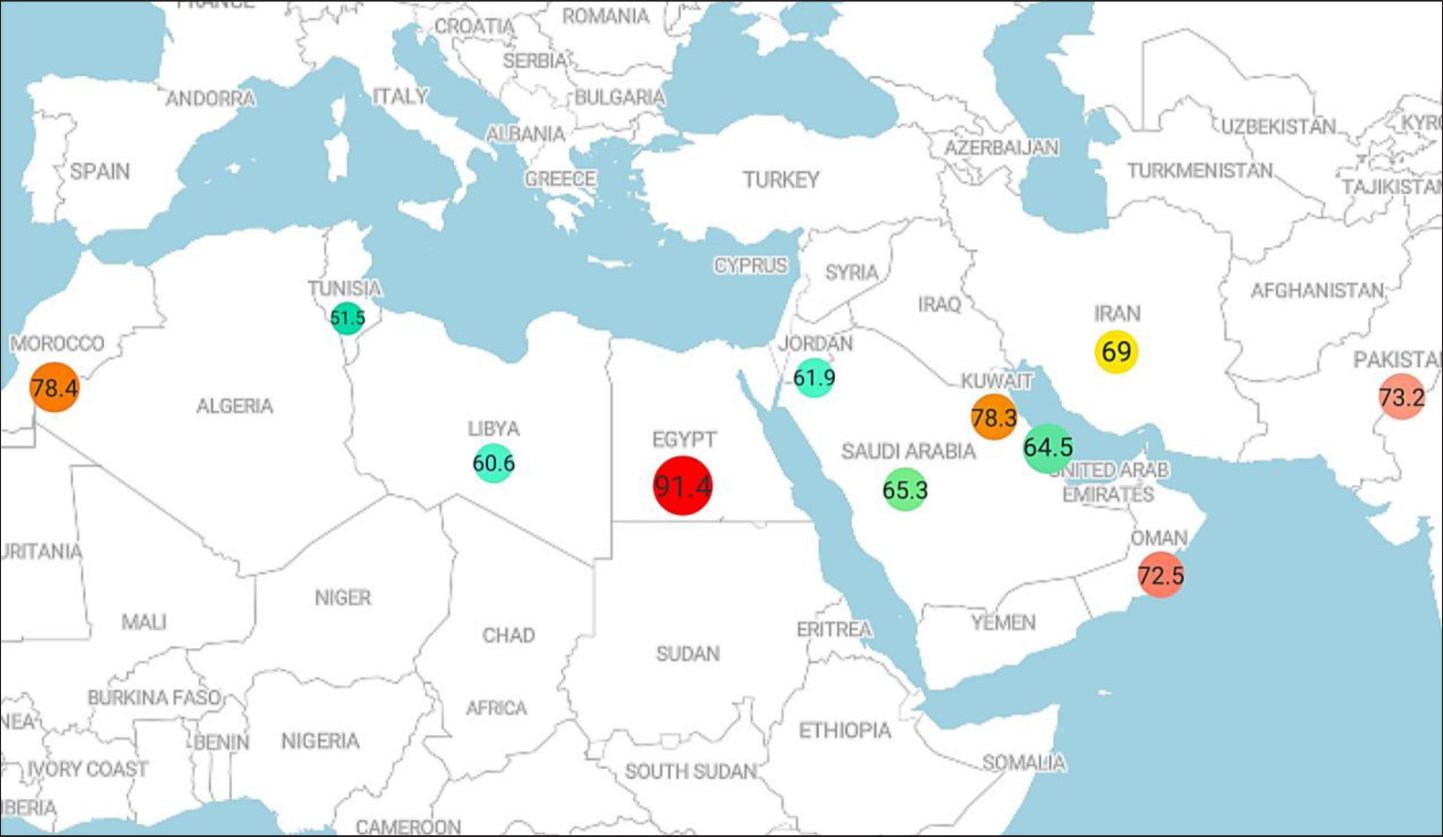
The five-year survival rate of breast cancer in EMRO countries.

Overall, the results of BC survival rate in 11 countries are reported in Table 2. The highest survival rates were reported for one (Morrocco), two (Iran), three (Egypt), four (Iran), five (Egypt), seven (Iran) and ten (Iran) years. Also, the lowest survival rates were reported for one (Bahrain), two (Tunisia), three (Tunisia), four (Jordan), five (Tunisia), seven (Tunisia) and ten (Bahrain) years. The results of five-year survival rate of BC in EMRO countries are shown in Figure 8.

**Table 2:** Result of meta-analysis of survival rate of breast cancer in EMRO base on each country and year of survival.

Country	Survival rate by year						

	1	2	3	4	5	7	10

**Bahrain**	84 (81.7–86.2)	NR	NR	NR	64.5 (59.5–69.5)	57.3 (48.5–66.1)	45 (35.7–54.3)
**Egypt**	NR	NR	96.4 (95.2–97.5)	NR	91.4 (89.6–93.1)	NR	NR
**Iran**	94 (92.4–95.6)	88 (81.4–94.6)	80.8 (77.5–84.1)	74.8 (64.4–85)	69 (64.5–73.4)	79 (74.1–83.8)	59.2 (49–69.4)
**Jordan**	92.5 (91.1–93.9)	81.6 (79.1–84.1)	72.4 (68.4–76.4)	67.5 (64.7–80.2)	61.9 (57.1–66.7)	NR	NR
**Kuwait**	NR	NR	83.2 (79.3–87)	NR	78.3 (73–83.7)	NR	NR
**Libya**	NR	NR	NR	NR	60.6 (53.2–68)	NR	NR
**Morocco**	97.1 (95.7–98.4)	NR	85 (76.6–93.4)	NR	78.4 (74.2–82.7)	NR	NR
**Oman**	NR	NR	NR	NR	72.5 (61.7–83.2)	NR	NR
**Pakistan**	NR	NR	NR	NR	73.2 (69.7–76.7)	NR	54 (51.2–56.8)
**Saudi Arabia**	NR	NR	NR	NR	65.3 (58.4–72.1)	NR	55 (49.5–60.5)
**Tunisia**	NR	49.1 (31.1–67.1)	33.7 (19.3–51.2)	NR	51.5 (46.8–56.1)	50 (46.4–53.6)	NR
**Overall**	93.6 (92.3–95)	85 (79.9–90)	79.8 (76–83.7)	72.6 (65–80.2)	69.2 (66.5–72)	62.1 (41.8–82.3)	55.5 (49.3–61.8)

#### Meta regression

**Figure 9:**
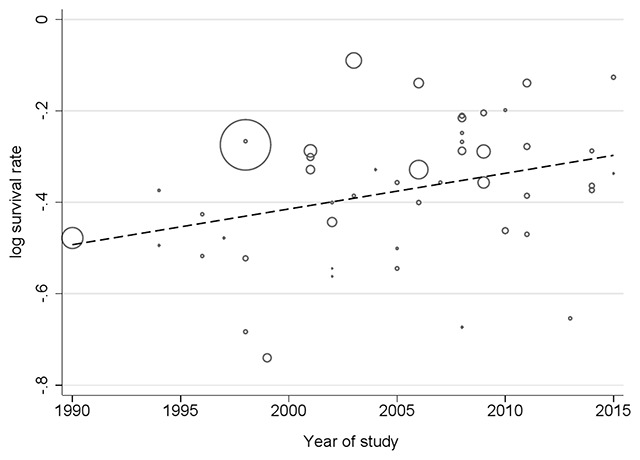
Result of meta-regression for five-year survival rate based on year of study.

Results of meta-regression illustrated a significant relationship between the year of publication and the five-year survival rate. Thus, the year of conducting study may be a cause of variability in the results of five-year survival rate (Reg Coefficient = 0.022, p < 0.001). However, the association for one-year survival rate was not statistically significant (Reg Coefficient = –0.010, p = 0.084). According to the results, an increasing survival rate across the study period was observed (Figure 9).

#### Publication bias

**Figure 10:**
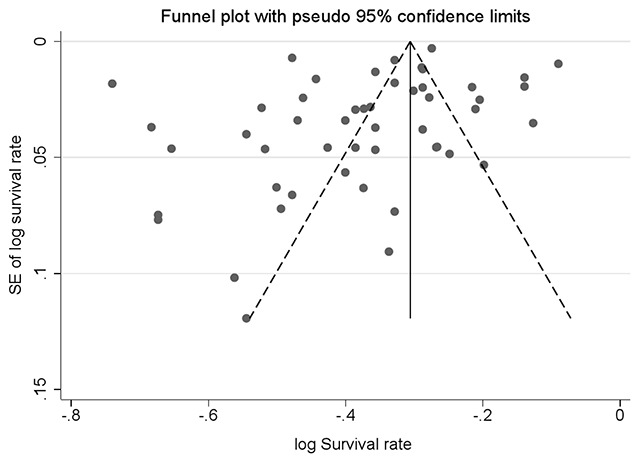
Funnel plot of standard error by point estimate for assessment of publication bias.

Finally, we created funnel plots to explore the possibility of publication bias, yet the results of Egger’s test were not evident of this bias (bias: –2.62, 95% CI = –5.30–0.05; P = 0.055). So, the most published articles on this subject should be considered (Figure 10).

## Discussion

The aim of this meta-analysis study was to estimate the survival rate of BC in different periods) 1, 2, 3, 4, 5 and 10 years (for EMRO countries. The overall survival rates were 93.9, 85.8, 79, 72.6, 69.2 and 55.5%, respectively. Also, the results of this study showed the distribution of survival rates in terms of HDI, with higher survival rates observed in countries with a high HDI. Estimates of survival in this study indicate that about 50% of BC patients die before 10 years. Several behavioral and environmental factors are associated both with the incidence and death rate of breast cancer. Therefore, the survival rate of patients between countries in the world as well as among countries in one region has a wide range. For example, in this study, the survival rate of 5 years in the evaluated countries varied from 51.5 in Tunisia to 91.4% in Egypt. In the study of the survival process in four European countries, it is shown that over the years 2000 to 2004, the survival rate of five years for Spain, Belgium and Portugal was 82% and for France, it was 86%.

In a meta-analysis study conducted in Iran, the five-year survival rate estimated from 11 papers was 67.7%, while this survival rate was 49% in Malaysia. Of course, comparing the results of this meta-analysis with other studies should be done with caution due to the method in which the study is performed and the sample size. The results of this meta-analysis—which showed that the highest 10-year survival rate for countries with high human development index is high—are consistent with previous studies, suggesting that low socioeconomic status is associated with delayed detection of cancer and lower survival rates.

Studies of economic inequality both within and between countries have depicted that the possible reasons for delay in diagnosis can be contributed to women’s access to health services and knowledge, and also attitudes toward cancer [12,13,14,15].

Screening programs are another issue that may have caused this relationship, so that informed and educated women are more likely to participate in the screening program. On the other hand, studies have shown a positive correlation between the HDI level and the incidence of BC. People in countries with a high HDI are associated with multiple cancer risk factors, and screening as well as diagnostic programs in these countries can be the reason for the high reported incidence of cancer. Studies have also shown that the ratio of death to incidence of BC is inversely related to HDI. The reason for this reverse correlation is related to health behavior after the diagnosis of cancer, since people in this countries have better access to health care. Also, coverage of health insurance in these countries can be another reason for this relationship.

The analysis of sub-groups showed that the highest 10-year survival rate was in Iran (59.2%), and was lowest in Bahrain (45%). A meta-analysis study on the survival of BC in Iran reported similar levels of survival rate of 58.1%. The amount of difference in reported rates can be related to the year of the study and the number of analyzes. Although in the countries of the EMRO, the highest 10-year survival rate was in Iran, this differs significantly from that of the European countries. Other study, that compares the survival rate of BC in Iranian patients with British Columbia patients, has shown that 30% of Iranian patients are diagnosed in the early stages of the disease, compared to 70% of British Colombia patients. On the other hand, the lowest 10-year survival rate occurring in Bahrain may be due to many reasons. In Arabic countries such as Bahrain, the age of catching BC is lower in comparison with Western countries, which can affect the survival of patients. One study conducted to determine the quality of life of patients with BC in Bahrain has also shown that the quality of life of Bahraini women is lower than that of the United Arab Emirates. Another study aimed at examining the epidemiology of BC in Bahrain showed that about half of the registered patients had an unknown grade and stage of the disease. In addition, a low percentage of patients were identified based on the screening program.

### Strengths and limitations

This study contained some limitations. The type and quality of the design studies, the sample size and the difference in the number of studies in the evaluated countries can be factors that affect the results. Regarding the high heterogeneity in studies, it was attempted to analyze results based on two main sources, the country and the level of HDI. The results of this meta-analysis, as compared to previous ones, can provide an acceptable estimation of the survival rates of patients in the region, such that they are useful for prevention and treatment programs.

### Recommendations for future research

According to the results of this study, estimating the survival rate of BC requires more extensive studies at the level of other countries, especially in West Asia. As most studies examined in this study were conducted in Iran and Saudi Arabia, estimates are somewhat incorrect. Another suggestion is a study of the survival of BC in patients who metastasized, which was not our study goal, and is an important issue in clinical decision-making and the continuation of treatment.

## Conclusions

Evidence suggests that about half of the patients in this area would die before 10 years survival, which is different from more developed countries. Also, high survival rates are associated with high human development index, which can help health policy-makers to better predict the outcomes of patients.

## Additional File

The additional file for this article can be found as follows:

10.5334/aogh.2521.s1 Appendix 1. Newcastle-Ottawa Quality Assessment Form.
