# Supplementary material for: Survival Rate of Breast Cancer in Eastern Mediterranean Region Countries: A Systematic Review and Meta-Analysis
Source: Ann Glob Health. 2019 Dec 4;85(1):138. doi: 10.5334/aogh.2521 (PMC6896839; doi:10.5334/aogh.2521)
Supplement: Appendix 1. — Newcastle-Ottawa Quality Assessment Form. [file agh-85-1-2521-s1.pdf]

Appendix 1. Newcastle-Ottawa Quality Assessment Form

Author (year)	Selection	Comparability	Outcome	Total	Quality
Al-Idrissi, 1992	2	1	2	5	Fair
Ibrahim, 1998	2	1	1	4	Fair
Fakhro, 1999	3	1	2	6	Good
Ahmed,2002	3	1	2	6	Good
Al Moundhri,2004	2	2	2	6	Good
Motawy, 2004	3	2	2	7	Good
Vahdaninia, 2004	3	1	2	6	Good
Ghavam-Nasiri, 2005	2	1	2	5	Fair
Ibrahim, 2005	2	1	2	5	Fair
Ravichandran, 2005	3	1	3	7	Good
Babaei, 2005	2	1	2	5	Fair
Rajaeifard, 2005	3	1	3	7	Good
Mousavi, 2006	2	1	2	5	Fair
Akbari, 2006	2	1	3	6	Good
Khanfir, 2006	2	2	2	6	Good
Yaghmaei, 2008	2	2	2	6	Good
Heydari,2009	2	2	2	6	Good
Rezaianzadeh, 2009	2	1	3	6	Good

Sadjadi, 2009	2	1	2	5	Fair
Abahssain, 2010	4	1	3	8	Good
Arkoob, 2010	2	2	2	5	Good
El Mongy, 2010	3	1	3	7	Good
Al-Moundhri, 2011	2	1	2	5	Fair
Fouladi, 2011	2	2	2	6	Good
Hamdan, 2011	3	1	3	7	Good
Tarawneh, 2011	3	1	3	7	Good
Movahedi, 2012	2	1	3	6	Good
Rahmani, 2012	2	1	3	6	Good
Rais, 2012	2	1	2	5	Fair
Vostakolaei, 2012	2	1	2	5	Fair
Ziaei, 2013	2	2	2	5	Good
Haghighat, 2013	2	1	2	5	Fair
Fallahzadeh, 2014	4	1	3	8	Good
Fayaz, 2014	4	1	3	8	Good
Sedehi, 2014	2	1	2	5	Fair
Fazeli, 2014	2	2	2	6	Good
Hamadeh, 2014	2	1	3	6	Good
Faradmal, 2014	2	2	2	6	Good
Karimi, 2014	2	1	2	5	Fair

Baghestani, 2015	2	1	2	5	Fair
El Mistiri, 2015	2	2	2	6	Good
Jamshed, 2015	2	1	2	5	Fair
Mahmood, 2015	2	1	2	5	Fair
Rampisheh, 2015	3	2	3	8	Good
Payandeh, 2015	2	2	2	6	Good
Rejali, 2015	2	1	2	5	Fair
Derkaoui, 2016	2	2	2	6	Good
Faradmal, 2016	3	2	2	7	Good
Kumar, 2016	2	2	2	6	Good
Mechita, 2016	2	2	3	7	Good
Rahimzadeh, 2016	2	2	2	6	Good
YektaKooshali, 2016	2	2	2	6	Good
Bakhshi, 2017	2	2	2	6	Good
Davoudi Monfared,2017	2	2	2	6	Good
El Amine Elhadj, 2017	2	1	2	5	Good
Hosseinpour Feizi, 2017	2	2	2	6	Good
Najafi, 2017	2	2	2	6	Good
